# Functional outcome and satisfaction with a “self-care” protocol for the management of mallet finger injuries: a case-series

**DOI:** 10.1186/s13032-014-0021-y

**Published:** 2014-12-10

**Authors:** Katriona Brooksbank, Paul J Jenkins, Iain C Anthony, Alisdair Gilmour, Margaret P Nugent, Lech A Rymaszewski

**Affiliations:** Department of Orthopaedic Surgery, Glasgow Royal Infirmary, 84 Castle Street, Glasgow, G4 0SF UK

**Keywords:** Mallet finger, Service redesign, Self-care, Fracture clinic redesign, Extensor tendon

## Abstract

**Background:**

Mallet finger injuries are usually successfully treated non-operatively with a splint. Most patients are reviewed at least twice in a clinic after the initial presentation in A&E. A new protocol promoting “self-care” was introduced at our institution. Patients were provided with structured verbal and written information, and given access to a telephone helpline.

**Methods:**

A prospective electronic patient record was used to identify all patients who presented to the emergency department with a mallet finger with a minimum six month follow-up. A satisfaction and patient reported outcome measure was administered via a postal questionnaire. The response rate was 36/47 (77%).

**Results:**

The median QuickDASH score was 2.3 (IQR 0 to 4.6). All patients were satisfied with the treatment plan provided. Nine used the helpline and all were satisfied with information given. Although 13 patients reported some extensor lag, or bump, they had no functional limitation. Seven patients were reviewed by the general practitioner or other clinicians during their treatment period for issues such a skin care, splint size changes or sickness certification. Five were subsequently reviewed at the end of their treatment period in a clinic at their request, or their general practitioner, but did not require further surgical intervention.

**Conclusions:**

Self-care for mallet finger injuries, with adequate patient information and telephone back-up, leads to acceptable functional results and satisfaction.

Level of evidence: III

## Introduction

The “mallet finger” injury is usually caused by forced hyperflexion of the distal interphalangeal joint (DIPJ), disrupting the terminal extensor tendon, or causing a fracture at the tendon insertion [1]. The majority of injuries occur to the dominant hand, in the ulnar sided digits [2].The aim of treatment is to achieve tendon healing without elongation, and therefore long-term extensor lag. The majority of simple and complex injuries can be satisfactorily managed with static splintage of the DIPJ [3]. The main determinant of treatment outcome is compliance with treatment [4]. A very small minority of injuries that combine a fracture with DIPJ subluxation may benefit from early identification and operative management. A long term follow-up found excellent functional outcome that was independent of extensor lag, bony involvement or the development of radiological DIPJ osteoarthritis [5].

The injury is usually initially diagnosed and managed by an emergency department (ED) or minor injuries unit (MIU) prior to review in an orthopaedic fracture clinic. This review is used to confirm the diagnosis, identify the small subgroup that may benefit from operative management and repeat information about the treatment plan. In the majority of cases no change is made to the treatment plan, making this routine orthopaedic assessment unnecessary and inefficient [3].

**Figure 1:**
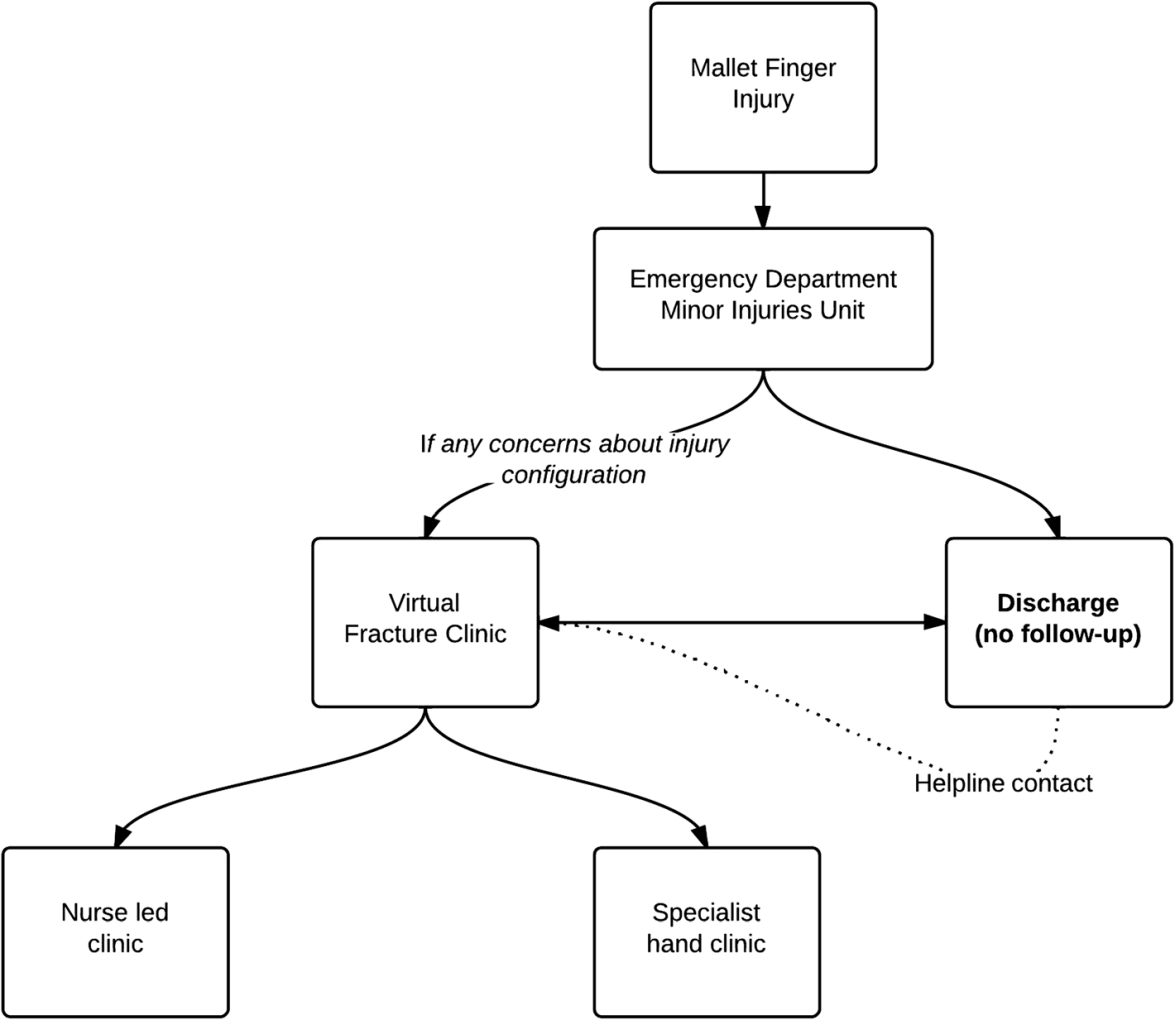
**Diagram of the referral process to virtual fracture clinic and specialist hand clinic.**

The fracture clinic service at our institution has been redesigned through collaboration and consensus between the ED and Orthopaedic clinicians [6]. The aim of this redesign was to provide a safe and effective service that minimised unnecessary review. It promoted “self-care” where appropriate by giving information and providing back-up. Patients with a range of simple, stable injuries that have well reported good outcomes with non-operative treatment are completely managed by the ED or MIU and no routine follow-up is arranged [7] (Figure 1). Verbal information is backed up by leaflets and an open access helpline. Mallet finger injuries have been managed in this way since 2011. Where the injury is complicated by a bony component or joint subluxation, the ED can refer the patient for “virtual review” in a regular multidisciplinary virtual fracture clinic (VFC). At this, an orthopaedic consultant reviews the patient’s history and radiographs. Following this review, all patients are phoned by a senior nurse and either discharged or appointed at the right time to see the appropriate specialist.

The primary aim of this study was to investigate the patient reported functional outcome one year following mallet finger injuries treated with this new protocol. The secondary aim was to investigate their satisfaction with the process.

## Methods

Patients with a mallet finger, presenting to our ED or MIU, between October 2011 and October 2012, were retrospectively identified using an administrative database (EDIS). There were 47 eligible injuries. The information in the leaflet included advice on continuous use of the splint and skin hygiene care.

Research ethics approval was not required as this study was defined as an audit of service evaluating satisfaction and outcome. Treatment was carried out according to accepted standards of care. A postal survey was sent to these patients. Patient reported functional outcome (PROM) was assessed using the QuickDASH score [8]. A higher value of this score indicated greater disability. General health was measured using the EQ-5D-5L score [9]. This was converted to a single health quality index using the UK value set. The separate EQ-5D visual analogue scale (VAS) was also measured. A five-level Likert scale (strongly agree, agree, neither agree or disagree, disagree strongly disagree) was used to assess satisfaction with the written information leaflet. Patients were asked the question, “Were you satisfied with the information provided regarding the treatment plan for your injury?”. This has been used in previous studies in our unit [7]. Patients were also asked whether they had used the helpline number, and if yes, they were asked if they were satisfied with experience using a binary scale (yes/no). They were asked whether they had attended their general practitioner with regard to their injury. A second postal satisfaction survey was sent three months later to non-responders, followed by a further attempt via telephone.

36 (77%) out 47 patients could be contacted. There were 8 (22%) women and 28 (78%) men with a mean age of 48 years (range 14 – 73 years). The mean follow-up time was 322 days (SD 122 days). There were three (8%) with an associated bony injury. Ten (28%) patients presented over seven days after their injury. Two patients were referred to the VFC at the time of their initial presentation. One was offered specialist review, but they only attended at a later stage. There was no significant difference between those contacted and those lost to follow up (LTFU) in terms of age (p = 0.081; Mann–Whitney U) and gender (p = 0.474, female vs male in LTFU group OR 1.94, 95% CI 0.51 to 7.48). None of the patients who were lost to follow-up had bony injuries on radiographic review. All available radiographs were reviewed by the senior author to examine the presence or absence of joint subluxation. None of those with a bony injury, treated with this protocol, had joint subluxation. During the study period there was one patient with a bony mallet injury and joint subluxation, who was treated with surgical management with a dorsal blocking wire technique. This patient was referred via the virtual fracture clinic the hand clinic, where they were identified for surgical management. Four of these patients had been accidently referred to the virtual clinic, rather than undergoing a virtual discharge from the emergency department. These four patients were “virtually discharged” after discussion at the VFC.

### Statistical analysis

The QuickDASH score and EQ-5D were not normally distributed. The central tendency and dispersion were therefore reported as a median and interquartile range. The outcome was compared between those with and without associated fracture, delayed presentation and gender, using the non-parametric Mann Whitney U test. Satisfaction with the process was reported using simple proportions.

## Results

**Figure 2:**
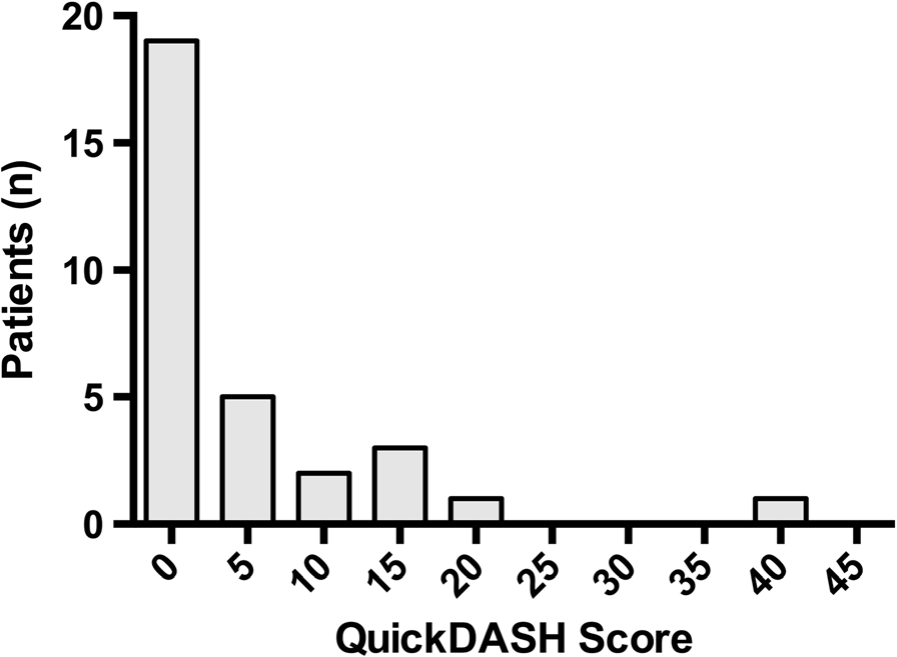
**Histogram of QuickDASH score at final follow-up.**

At final follow-up the median QuickDASH score was 2.27 (IQR 0 to 4.55) (Figure 2). The median EQ-5D VAS score was 90 (IRQ 75 to 90). The median EQ-5D health index was 0.88 (IQR 0.84 to 1.00). There were three patients with a QuickDASH score greater than 15. One of these patients had pre-existing joint pain that accounted for their score, but was still satisfied with the outcome of their injury. The second patient had their injury initially missed by the ED department, but returned the next day. They did not report any final extensor lag. The final patient presented to the ED department eight weeks after the injury and was referred via the VFC to the specialist hand clinic for review and management.

**Figure 3:**
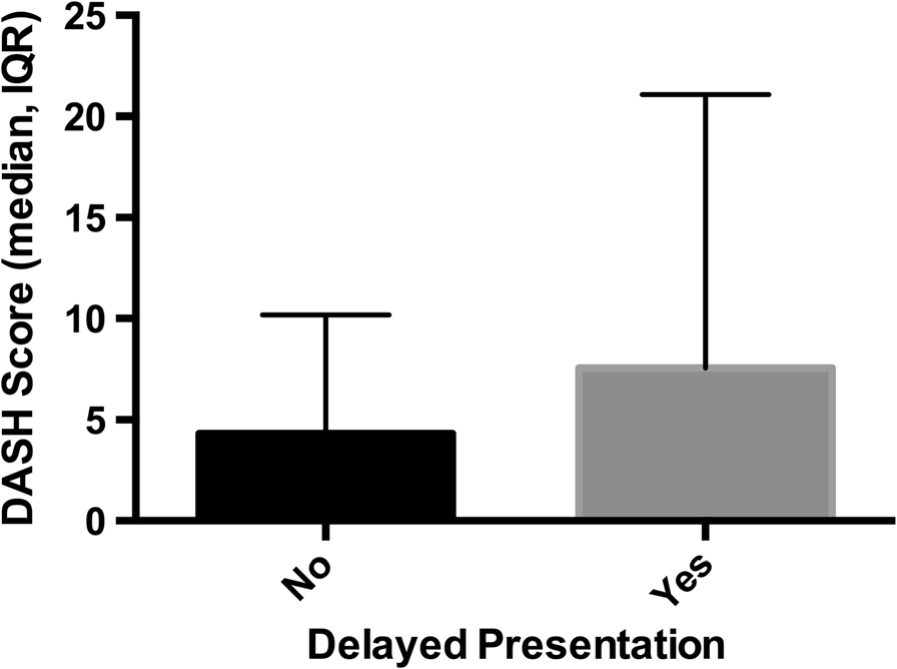
**Comparison of QuickDASH score in those with immediate versus delayed presentation (median, interquartile range).**

There were no significant differences in functional outcome between those with or without a bony component to their injury (MWU p = 0.127). There was no difference between those who presented immediately and those who presented late (MWU p = 0.928) (Figure 3).There was no correlation of age with QuickDASH (p = 0.415), EQ-5D VAS (p = 0.119) or EQ-5D health index (p = 0.108).

All patients were satisfied with the process and information provided; 20 patients (56%) were very satisfied with the process and information provided and 16 (44%) were satisfied. There was no dissatisfaction. Two patients (6%) commented that they would have liked some advice on suitable exercises to be done once the splint was removed and one patient (3%) would have liked a follow up appointment for reassurance.

The helpline was used by nine patients (25%); all (100%) were satisfied with the experience. Of those who responded to the survey, seven (19%) had visited another doctor, usually their general practitioner, during the initial recovery period. One of these required certification for absence from work. Six sought medical advice for skin problems, blisters and a change of splint size due to finger swelling. Five patients (14%) were subsequently (> 6 weeks) seen at consultant-led specialist orthopaedic clinics and two were referred on to the specialist hand physiotherapy service for exercises to treat stiffness. To access the specialist orthopaedic hand team one patient visited their GP two months after the injury, at the time of splint removal. One patient with an associated bony injury had been discussed at the VFC and an appointment had made at the hand clinic. They did not however attend immediately, but represented late, after three months and were managed conservatively. Two patients used the helpline number after two and five months respectively and were offered appointments. Both these patients had a dorsal prominence at the DIPJ and minor extensor lag. One patient re-attended the MIU early and was referred to the specialist clinic at that stage, no further action was necessary, other than reiteration of the splint care advice. No patients required secondary surgery during the study period.

## Discussion

This study of mallet finger patients shows that acceptable levels of patient satisfaction can be achieved using our new protocol for direct discharge from the ED or MIU with standardised advice. The dissatisfaction rate was in line with reported literature for mallet finger injuries and therefore similar to what would have been achieved with a traditional fracture clinic review system for all fracture patients [5,10].

A Cochrane review examined the interventions for treatment of mallet finger, specifically at four randomised controlled trials (RCT), involving a total of 278 participants with 283 mallet finger injuries and concluded that no difference could be detected between different splint types [11]. They reiterated that compliance with treatment and length of immobilisation were the most important determinant of outcome. The outcome of non-operative treatment appears to be good, regardless of extensor lag, bony bump, DIPJ arthritis or joint subluxation.

The group of patients in this study reported a low median QuickDASH score, good general health and satisfaction with the clinical outcome and the process. For those patients who did rate themselves as having pain, depression or poor health this was not attributed to their mallet finger injury but other long-standing health concerns. Universal satisfaction was reported for the standardised advice leaflet given to mallet finger patients on discharge from ED. Of those respondents who used the helpline number included in the advice leaflet all were satisfied with the service received. Patients were still able to access the helpline services and be recalled for specialist orthopaedic review once their splint had been removed, allowing both initial and longer term complications to be dealt with as appropriate. Access through GP referral was also available for patients who were still experiencing problems once the initial splinting period was complete. It must be stressed that this process has several safeguards to ensure patient safety, with adequate early education and an open door policy if recovery fails to meet expectations.

Overall, although the fracture clinic redesign has been primarily patient focused, to reduce unnecessary hospital visits, inconvenience, expense and time off work, the same principles have considerable potential to improve efficiency throughout the healthcare system. In clinic, extra time can be given to considering more complex cases. It was outwith the scope of this study to quantify the economic savings of this process to the health service. In addition, the redesign has been demonstrated to have no adverse effects on the ED process [6].

This study benefits from a reasonable rate of follow-up and the use of modern patient reported outcome measures combined with a satisfaction questionnaire. Patients were identified retrospectively from a prospectively collected administrative database. The diagnosis of mallet finger is a common one to be made by the ED or MIU and there is little diagnostic doubt. We are therefore confident that we have fully identified these patients over our study period. As a result of this study we plan to modify the information leaflet to provide information about exercise that can be performed after splint removal. In particular we plan to highlight issues of skin care, with diagrams demonstrating the correct procedure for removing and replacing the splint. This could additionally be provided using new technology such as online streamed media. A common issue is need for secondary contact when the initial swelling has subsided, to provide a smaller splint. A second, smaller splint could be supplied to all patients for application in this scenario. This may reduce the number of secondary contacts and facilitate self-care.

## Conclusion

In conclusion, this study demonstrates that a protocol which encourages self-care and eliminates regular review resulted in good function and excellent satisfaction. These results are at least equivalent to those reported in studies with regular follow-up. The introduction of this protocol can reduce unnecessary review and use healthcare resources more efficiently.
